# Skin disorders in free-ranging Chilean dolphins (*Cephalorhynchus eutropia*) from Biobío, Chile

**DOI:** 10.29374/2527-2179.bjvm007625

**Published:** 2025-11-10

**Authors:** Felipe Oyarzún-Cordova, Marie-Francoise Van Bressem, Camila Calderón-Quirgas, Gierke Medina-Rojas, Daniel Lagos-Alister, Heraldo V. Norambuena, Andrea Cisterna-Concha

**Affiliations:** 1 Programa de Magister en Gestión Ambiental, Facultad de Ingeniería, Universidad San Sebastián, Concepción, Chile.; 2 Centro de Estudios de Mastozoología Marina. Concepción, Chile; 3 Cetacean Conservation Medicine Group, Peruvian Centre for Cetacean Research, Museo de Delfines. Lima, Perú; 4 ProDelphinus, Lima 18, Peru; 5 Centre d’Éducation et de Recherche de Sept-Îles, Québec, Canada.; 6 Graduate program in Oceanography, Faculty of Natural Sciences and Oceanography, Department of Oceanography, University of Concepción, Concepción, PO Box 160-C, Chile.; 7 Facultad de Ciencias Veterinarias, Universidad de Concepción, Concepción, Chile.; 8 Centro Bahía Lomas, Facultad de Ciencias, Universidad Santo Tomás, Chile.

**Keywords:** skin lesions, Chilean dolphin, tattoo skin disease, pale skin patches, orange hues, lesões cutâneas, golfinho chileno, doença cutânea tipo “tattoo”, manchas cutâneas pálidas, coloração alaranjada

## Abstract

Skin disorders reports in cetaceans are increasing worldwide and may reflect individual or population health. This study describes the occurrence and distribution of skin disorders in Chilean dolphins *Cephalorhynchus eutropia* from Gulf of Arauco, Chile, based on photographic records collected during a summer 2024 expedition. A total of 685 photos were reviewed, with 39 meeting quality criteria and 16 showing identifiable skin lesions. Of the 37 dolphins photo-identified, 17 (45.95%) presented lesions. Tattoo skin disease (TSD) was diagnosed in 29.73% of individuals, with one animal showing signs of healing. Pale skin patches (PSP) and orange hues were also observed, with a prevalence of 10.81% and 5.41%, respectively. Lesions were primarily located on the thorax and lumbar regions and varied in size. This is the first report of TSD in Chilean dolphins from the Gulf of Arauco. Although the aetiology of PSP and orange hues remains uncertain, they may be associated with environmental factors such as salinity and diatom colonization. Given the conservation category of this endemic species, continuous monitoring of skin conditions and environmental parameters is recommended to understand the health implications and design conservation strategies.

## Resumo

Os relatos de distúrbios cutâneos em cetáceos têm aumentado em todo o mundo e podem refletir o estado de saúde individual ou populacional. Este estudo descreve a ocorrência e a distribuição de distúrbios cutâneos em golfinhos chilenos (*Cephalorhynchus eutropia*) do Golfo de Arauco, Chile, com base em registros fotográficos coletados durante uma expedição realizada no verão de 2024. Um total de 685 fotografias foi analisado, das quais 39 atenderam aos critérios de qualidade, e 16 apresentaram lesões cutâneas identificáveis. Dos 37 golfinhos fotoidentificados, 17 (45,95%) apresentaram lesões. A doença cutânea do tipo “tattoo” (Tattoo Skin Disease – TSD) foi diagnosticada em 29,73% dos indivíduos, com um animal mostrando sinais de cicatrização. Também foram observadas manchas cutâneas pálidas (Pale Skin Patches – PSP) e coloração alaranjada, com prevalências de 10,81% e 5,41%, respectivamente. As lesões localizaram-se principalmente nas regiões torácica e lombar e variaram em tamanho. Este é o primeiro registro de TSD em golfinhos chilenos do Golfo de Arauco. Embora a etiologia das PSP e da coloração alaranjada permaneça incerta, essas alterações podem estar associadas a fatores ambientais, como salinidade e colonização por diatomáceas. Considerando a categoria de conservação dessa espécie endêmica, recomenda-se o monitoramento contínuo das condições cutâneas e dos parâmetros ambientais para compreender as implicações sobre a saúde e subsidiar estratégias de conservação.

## Introduction

Miscellaneous skin disorders have been documented in several species of cetaceans worldwide, including odontocetes and mysticetes, at an increasing rhythm during the last 30 years (Luciani et al., 2022; Sanino et al., 2014; Segura-Göthlin et al., 2023; Van Bressem & Van Waerebeek, 1996; Van Bressem et al., 2007, 2009, 2022, 2024). They have been included in visual health assessment of free-ranging dolphins and whales, providing an insight into the viruses, fungi and parasites affecting them (Hanninger et al., 2023; Kautek et al., 2019; Minton et al., 2022; Van Bressem et al., 2007). They include a variety of lesions whose aetiology, except for tattoo skin disease (TSD), erysipelas, and lobomycosis, is usually unknown (Bertulli et al., 2012; Kautek et al., 2019; Neves et al., 2024; Toms et al., 2020; Van Bressem et al., 2022, 2024). In Chile, the presence of dermopathies has been described in both Peale’s dolphin *Cephalorhynchus australis* (Peale, 1848) and Chilean dolphin *Cephalorhynchus eutropia* (Gray, 1846), (Society for Marine Mammalogy, 2025). TSD is a disease caused by poxviruses (CePV) that affect cetacean worldwide (Van Bressem & Van Waerebeek, 1996; Van Bressem et al., 2022). It is characterized by gray or black cutaneous lesions with rounded margins and a characteristic stippled pattern that are distributed on the whole body or localized to certain areas, such as the head in Burmeister’s porpoises *Phocaena spinipinnis* (Burmeister, 1865) (Sanino et al., 2014; Van Bressem & Van Waerebeek, 1996). TSD affects all age classes, despite being rare in neonates and young calves, its prevalence increases in older calves and juveniles, likely because they have lost passive immunity (Powell et al., 2018; Van Bressem et al., 2009). Besides TSD, Sanino et al. (2014) reported on pale skin patches (PSP), focal skin disease (FSD), and skin linear anomalies (SLA) in Chilean dolphins from the Añihué Reserve, Región de Aysén, Chilean Patagonia.

The Chilean dolphin is the only cetacean species endemic to Chile (Yáñez, 1948) and it is currently classified as Near Threatened (NT) by the International Union for Conservation of Nature (IUCN) (Heinrich & Reeves, 2017). Two large populations have been observed, a northern one between Valparaíso (33.04°S) and the Chiloé Island (42.5°S) and a southern one between the Chiloé and Navarino Island (55.08°S; Aguayo-Lobo et al., 1998; Pérez-Álvarez et al., 2007). The northern population inhabits areas with open coastlines, bays, and estuaries while the southern population resides in highly fragmented inshore coastlines, channels, and fjords (Pérez-Alvarez et al., 2015).

The southern part of the Gulf of Arauco, Región del Biobío in Chile is one of the most important areas for this species, with an abundance estimated at 134 animals (Sepúlveda et al., 2020; Valenzuela, 2022). This region that receives great volume of fresh waters from large rivers, *e.g.*, Biobío River, and smaller ones such as the Tubul and Raqui rivers (Villagrán et al., 2023). It is characterized by seasonal coastal upwelling driven by the winds fuelled by the Humboldt current, resulting in high primary productivity (Iriarte et al., 2012; Masotti et al., 2018; Sobarzo et al., 2007). The waters of the Gulf have an average salinity of 34.14 psu, a temperature of 13.24 °C and dissolved oxygen levels of 4.85 ml/L. Heavy metals, nitrates, phosphates, and organic compounds were also detected in January 2022 in this area (Chile, 2022).

**Figure 1:**
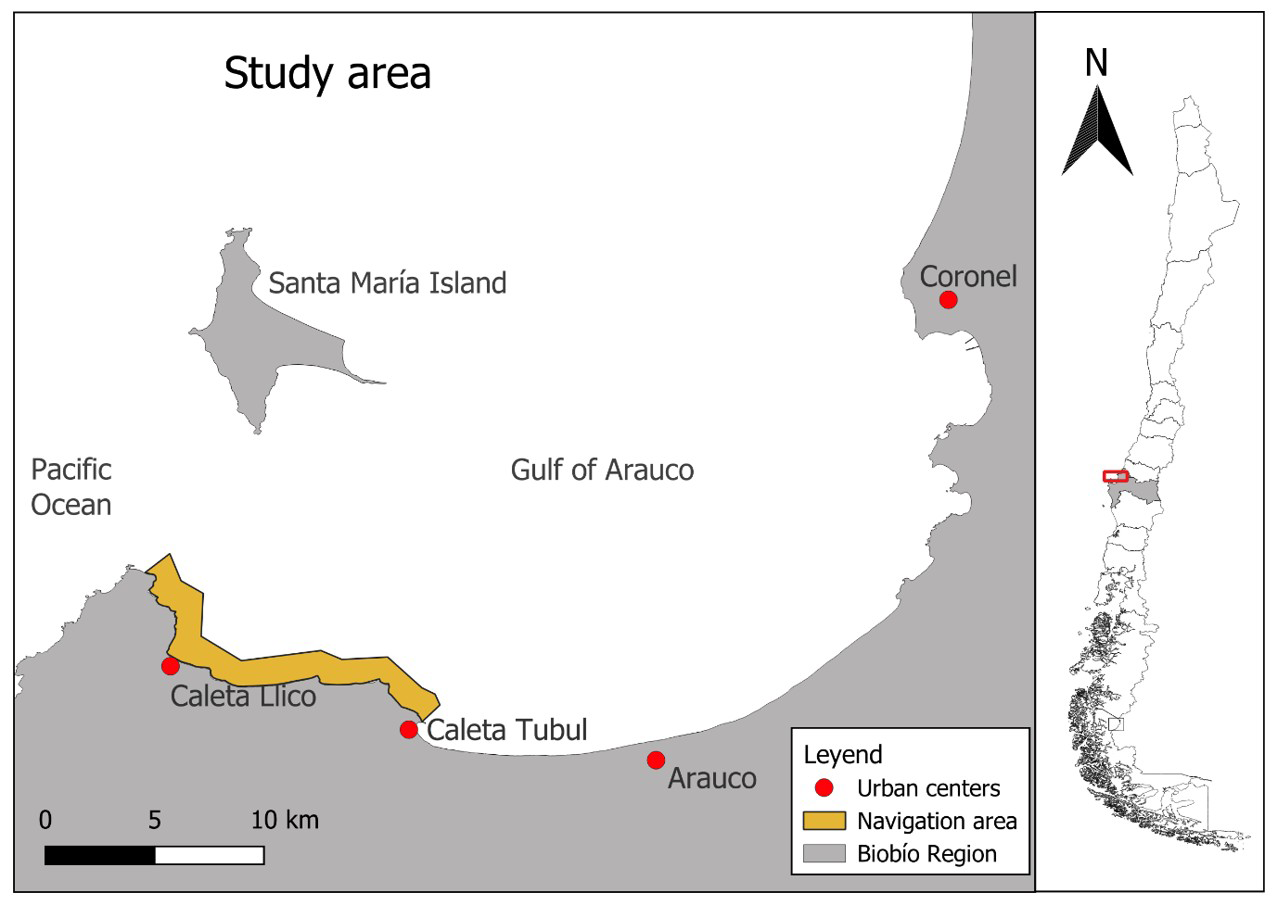
Study area of the Chilean dolphin *Cephalorhynchus eutropia* in Caleta Llico (37.19°S, 73.55°W), Gulf of Arauco, Región del Biobío, Chile. Red dots represent the urban centers in the southern part of the Gulf of Arauco while the highlighted yellow band corresponds to the navigation area where the specimens with skin lesions were recorded.

Gulf of Arauco is surrounded by several urban centers which, include small towns such as Caleta Llico and Tubul, as well as big cities such as Arauco and Coronel (Figure 1). It has been recently defined as an Important Marine Mammals Area (IMMA) by The International Union for Conservation of Nature (IUCN) and the Marine Mammals Protected Area Task Force (MMPATF) in 2023 (Marine Mammal Protected Areas Task Force, 2023). Besides being home to the largest known population of Chilean dolphins, the Gulf of Arauco also hosts large numbers of marine otters *Lontra felina* (Molina, 1782) and the otariids South American sea lion *Otaria byronia* (de Blainvillei, 1820) and South American fur seal *Arctocephalus australis* (Zimmermann, 1783) and is often visited by sei whale *Balaenoptera borealis* (Lesson, 1828), (Cisterna-Concha et al., 2023; Marine Mammal Protected Areas Task Force, 2023).

This study aims to report, for the first time, the presence of skin lesions in the population of Chilean dolphins inhabiting the Gulf of Arauco, an ecologically important and conservation-priority area for this species in Chile coast.

## Material and methods

During the austral summer of 2024, a scientific expedition was conducted to study the population of Chilean dolphins inhabiting Caleta Llico (37.19°S, 73.55°W), Gulf of Arauco, Región del Biobío, Chile. The fieldwork was led by the Centro de Estudios de Mastozoología Marina and aimed at obtaining photographic records for the individual photo-identification of dolphins in the area. Five marine transects were carried out over 18.9 hours of effort during January and February, using a Nikon D5300 with a 150-600 mm lens and a Canon EOS 7D with a 100-400 mm lens. In addition to the planned sampling effort, complementary photographic records obtained during a tourist excursion in February 2024 at the same location were incorporated into the analysis due to their relevance. While two trained observers were examining the images for photo-identification, they observed cutaneous lesions compatible with dermatological disorders previously reported in this species (Sanino et al., 2014), prompted further investigation.

Photographs were carefully selected on the basis of image quality quality, *i.e.,* sharpness, lighting, and contrast. Only those that allowed precise identification of the individual and a clear view of the lesions, either at original size or when zoomed in, were selected for the analysis. Out of 685 photographs examined, 39 met the quality criteria, and 16 displayed identifiable cutaneous lesions. These lesions were classified according to external characteristics and measured using Image J^®^ software version 1.54 (Schneider et al., 2012). They were categorized by size (small, medium, large, very large or unmeasured), following criteria adapted from Sanino et al. (2014) and Van Bressem et al. (2022). Additionally, the topographic location was noted as follows: head, thorax, pectoral fin, dorsal fin, lumbar region, flanks, caudal peduncle and fluke, as described by Sanino et al. (2014).

## Results

**Figure 2:**
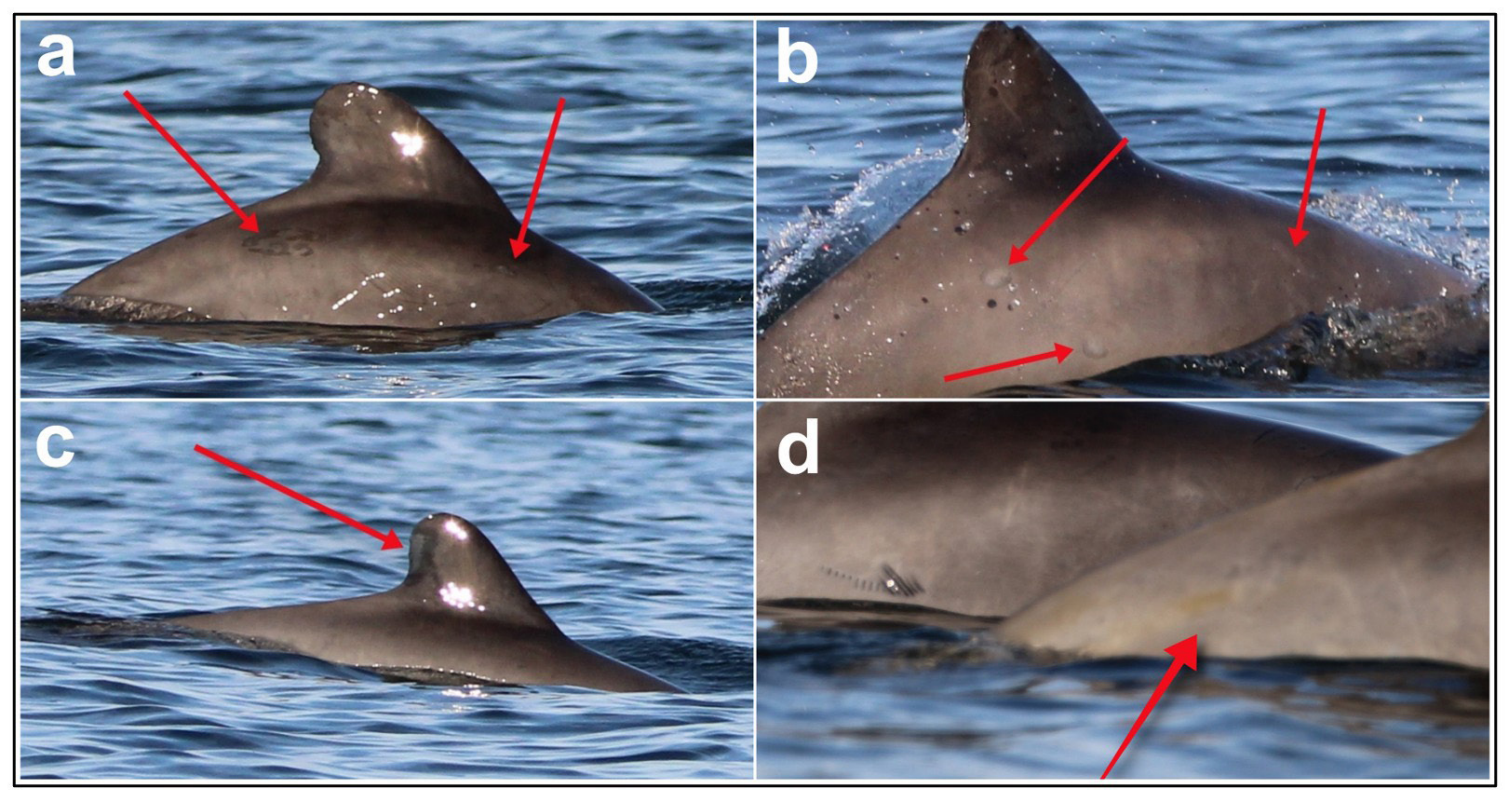
Skin lesions identified in Chilean dolphin *Cephalorhynchus eutropia* from Caleta Llico (37.19°S, 73.55°W), Gulf of Arauco, Región del Biobío, Chile. (a) Tattoo skin disease (TSD), (b) healing tattoo, (c) pale skin patches, and (d) orange hue.

During the days of fieldwork 37 animals were identified (Lagos-Alister, unpublished data). After selecting the photographs, it was possible to identify lesions in 17 photo-identified dolphins (Hupman et al., 2017). Eleven had skin lesions resembling tattoo skin disease (TSD) (29.73%), *i.e*., dark grey lesions with irregular or rounded and hyperpigmented margins (Table 1, Figure 2a). One animal (2.70%) also had healing tattoo skin lesions recognizable by a light grey oval lesion without a darker stippled outline (Table 1, Figure 2b). Small to very large lesions, numbered between one and three per animal, were mostly observed in the lumbar region and thorax. Medium sized pale skin patches (PSP) lesions numbering between one and two were found on the dorsal fin and thorax of four individuals (10.82%) (Figure 2c). Large and very large orange-hued areas were seen in the lumbar region of two other dolphins (5.41%) (Figure 2d). Altogether, the estimated prevalence of skin disorders for *C. eutropia* identified from Caleta Llico, Gulf of Arauco during January 2024 was 43.58%. The description of each type of skin lesion observed is provided in Table 1.

**Table 1:** Type of skin lesions observed in Chilean dolphin Cephalorhynchus eutropia from Caleta Llico, Gulf of Arauco, Región del Biobío in Chile, adapted from Gaydos et al. (2023), Sanino et al. (2014) and Van Bressem et al. (2022).

**Animal**	**Description**
Tattoo skin disorder (TSD)	Dark grey lesions with irregular or rounded and with hyperpigmented margins
Healing tattoos	Light grey oval lesion without the darker stippled outline as TSD
Pale skin patches (PSP)	Areas of opaque or translucent skin that seem to have lost its normal pigmentation
Orange hue	Skin disorder of orange color, often without delineated margins, covering the natural skin color over a pre-existing skin lesion

During fieldwork, no recaptures were recorded, and the evolution of skin lesions could not be evaluated. Other infectious diseases, such as lobomycosis and cutaneous erysipelas, were not detected in these dolphins.

## Discussion

Though TSD has already been visually diagnosed in Chilean dolphins from Añihue, (Sanino et al. 2014) this study is the first to report this skin disorder in the 115 Gulf of Arauco. Molecular diagnosis should be carried out on skin lesions sampled in dead dolphins to confirm the poxviral aetiology (Blacklaws et al., 2013; Luciani et al., 2022; Segura-Göthlin et al., 2023). The origin of PSP is unknown but could be related to oceanographic factors, such as salinity and oxygen levels (Boileau et al., 2024; Duignan et al., 2020; Sanino et al., 2014). Thus, Sanino et al. (2014) considered that PSP may result from sloughing of epidermis following long-term permanence of dolphins in quasi freshwater habitat. This should be further investigated considering the continuous input of river discharges from Tubul and Biobio rivers and the variable levels of salinity in the area (Vergara et al., 2024). Orange hues are skin disorders associated with the colonization by diatoms over a pre-existing lesion (Gaydos et al., 2023) and further research on its origin must be conducted.

Monitoring the population's environmental conditions and epidemiological surveillance is required to assess the evolution and dynamics of skin diseases and the influence of external factors on the cutaneous conditions. Since the Chilean dolphin is the only endemic cetacean of Chile and it is classified as Near Threatened (NT) by the IUCN (Heinrich & Reeves, 2017), the study of diseases that affect different populations is important for the development of public and/or private policies for its conservation.

## Conclusions

This study represents the first record of cutaneous lesions in the Chilean dolphin population from Caleta Llico, Gulf of Arauco in Chile, revealing an estimated prevalence of 43.58%. The most frequently observed skin lesions were consistent with tattoo skin disease (TSD), followed by pale skin patches (PSP) and orange-hued areas. These alterations were mainly located on the lumbar region and thorax, with sizes ranging from small to very large.

Although the diagnosis was based exclusively on visual evidence, the presence of TSD in this population suggests active circulation of poxviruses in the area. However, it is recommended that future studies incorporate histopathological and molecular analyses of skin tissues obtained from strandings or other sources, to confirm the aetiology of the observed lesions. Additionally, systematic long-term monitoring is advised to assess the progression of skin lesions and to investigate potential relationships between the incidence of dermopathies and local environmental factors.
